# Genetically predicted plasma metabolites mediate the relation between inflammatory factors and Meniere's disease

**DOI:** 10.1016/j.bjorl.2026.101772

**Published:** 2026-02-05

**Authors:** Jian Wang, Jian-Dao Hu, Jing Qian

**Affiliations:** The Affiliated People's Hospital of Ningbo University, Department of Otorhinolaryngology Head and Neck Surgery, Zhejiang Province, China

**Keywords:** Mendelian randomization, Inflammatory factors, Plasma metabolites, Meniere disease

## Abstract

**Objective:**

Emerging evidence suggests inflammation contributes to Meniere’s Disease (MD), a chronic vestibular disorder characterized by vertigo, hearing loss, and tinnitus. However, the causal role of Inflammatory Factors (IFs) and the mediating effects of Plasma Metabolites (PMs) in MD remain unclear. This study investigated bidirectional causality between IFs and MD and evaluated PMs as potential mediators.

**Methods:**

Using Genome-Wide Association Study (GWAS) summary data, we performed bidirectional Mendelian Randomization (MR) analyses to assess causal links between 91 IFs, 1,400 PMs, and MD. Single Nucleotide Polymorphisms (SNPs) genome-wide significant for IFs, PMs, and MD served as instrumental variables. Inverse Variance Weighting (IVW), MR-Egger, and weighted median methods were applied. Mediation analysis quantified PMs’ role in IF-MD associations. Sensitivity analyses (MR-PRESSO, leave-one-out) tested robustness.

**Results:**

Genetically predicted higher CCL23 levels reduced MD risk (IVW OR = 0.5757 per SD decrease; 95% CI 0.3679–0.9007; p = 0.0156). No reverse causality from MD to IFs was observed (IVW OR = 0.9919; 95% CI 0.9812–1.0028; p = 0.1448). Mediation analysis revealed PMs accounted for 14.6% of the protective effect of IFs on MD. Elevated IF levels correlated with increased PMs (IVW OR = 1.0788; 95% CI 1.0125–1.1494; p = 0.0192), while higher PMs reduced MD risk (IVW OR = 0.5444; 95% CI 0.3006–0.9859; p = 0.0448). Sensitivity analyses confirmed result consistency, with no significant pleiotropy.

**Conclusion:**

This study identifies CCL23 as a protective factor against MD, partially mediated by PMs. Findings underscore inflammation and metabolic pathways as potential therapeutic targets. Further validation in diverse populations and exploration of biological mechanisms are needed to advance MD treatment strategies.

**Level of evidence:**

5.

## Highlights

• MR Revealed IF→MD unidirectional causality, and PMs mediated 14.6% risk reduction.
• CCL23 is associated with the inflammatory/repair/stress mechanisms of MD.
• X-23639 is positively correlated with CCL23, or it may be a new marker.

## Introduction

Meniere's Disease (MD) is a chronic vestibular disorder characterized by recurrent dizziness, tinnitus, and sensorineural hearing loss in severe cases.^1^ First described by Prosper Meniere in 1861, its pathophysiology remained poorly understood until advances in medical research highlighted roles for genetic, environmental, and immunological factors.^2^ Affecting approximately 0.2% of the global population, predominantly middle-aged individuals, MD significantly impairs quality of life due to its impact on hearing and balance.^3^

While endolymphatic hydrops ‒ abnormal fluid accumulation in the inner ear ‒ was historically considered MD’s primary cause, emerging evidence underscores immune dysregulation, inflammation, and metabolic disturbances as key contributors.^4^ Notably, elevated inflammatory cytokines in the endolymphatic sac and serum of MD patients suggest inflammation’s pivotal role, prompting investigations into anti-inflammatory therapies.^5^ Concurrently, metabolites ‒ small molecules influenced by genetics, environment, and lifestyle ‒ have gained attention. Metabolomic studies reveal their involvement in modulating inflammatory responses, blood-labyrinth barrier permeability, and immune cell activity, potentially linking systemic inflammation to inner ear pathology.^6^^,^^7^ However, establishing causality between inflammation, metabolites, and MD remains challenging due to confounding factors in observational studies, such as reverse causality and comorbidities.^8^

Mendelian Randomization (MR), leveraging genetic variants as instrumental variables, offers a robust approach to infer causality. This study employs a bidirectional, two-sample MR framework to explore genetic associations between 91 Inflammatory Factors (IFs), MD, and 1400 Plasma Metabolites (PMs). We aim to determine: 1) Whether genetically predicted IF levels influence MD risk, 2) If MD causally affects IFs, and 3) Whether PMs mediate IF-MD relationships. By elucidating these pathways, our work provides novel insights into MD pathogenesis and identifies potential therapeutic targets.

Building on existing literature, this MR analysis clarifies the interplay between inflammation, metabolism, and MD. Our findings advance understanding of MD pathophysiology and emphasize the need to target inflammatory-metabolic pathways for therapeutic development, offering a foundation for future mechanistic studies.

## Methods

### Study design

The data we used in our study came from public sources and had been approved by the institutional review committees of relevant studies. So, we didn't need any more approvals. All the research results are shown in the article and its supplementary materials. In this research, we used the two ‒ pattern, biimmediateional Mendelian randomization way to study the two ‒ way causality between IFs and MD.^9^ In our study, we chose SNPs as instrumental variables.

### GWAS summary data sources

The data employed in this work are all from open sources, and the participators in GWAS are all European people. In a prior GWAS meta ‒ exploration encompassing 14,824 participators, 91 Inflammatory Factors (IFs) were identified to have a connection with genetic elements.^10^ To access the comprehensive statistical data of the whole- protein GWAS, one can visit https://www.phpc.cam.ac.uk/ceu/proteins and the EBI GWAS immediateory (where the registration numbers span from GCST90274758 to GCST90274848) for download. We combine these data with the whole ‒ disease genome ‒ wide relation study, aiming to uncover the functionality impacts of disease ‒ related variations. The data of MD were obtained from the GWAS catalog summary data source in https://www.ebi.ac.uk/gwas/studies/GCST90043830, covering 171 cases and 456,177 participators. As long as the individual's ICD code is [ICD10 H81.0 “MD”], it will be identified as MD cases. Summary statistics of 1400 PM ranks are derived from a series of large-scale GWAS meta-research.^11^ This research involved 8299 individuals in the CLSA cohort, including 1091 metabolites and 309 metabolites. In the initial literature, the specific particulars of the research and design, like pattern collection, the quality control procedure, and the reduction way, are elaborated in detail. Since all the GWAS data are sourced from diverse relations or organizations, there is no issue of pattern duplication.

### Instrumental variable selection and data harmonization

For our study, we picked SNPs that were very marked (p < 5 × 10^−8^). If there were no such super - marked SNPs for use as IVs, we chose SNPs with a lower marked rank (p < 5 × 10^-6^) as possible IVs.^12^ Then, we classified these SNPs according to linkage disequilibrium. We used a window size of 10,000 κb and a correlation coefficient (R²) less than 0.001. We got the linkage disequilibrium appraise from European patterns in the 1000 Genomes Project. If a SNP for a certain exposure wasn't in the outcome dataset, we found replacement (proxy) SNPs using linkage disequilibrium tagging. In the Mendelian randomization exploration, we take away palindromic and unclear SNPs from the IVs. We computed the F-statistic. We used the variance that SNPs illustrated for each exposure factor. The formula was [(N - K - 1)/K]/[R² /(1 - R² )], where K is the number of genetic variants and N is the pattern size. In the end, we got rid of weak instrumental variables, which were those with an F-statistic less than 10.

### Statistical exploration

We conducted the MR exploration employing R software (version 4.2.1, available at http://www.r-project.org) and the “Two ‒ Pattern MR” package (version 0.5.6).^13^ For the MR Pleiotropic Residual and Outlier Measurement (MR-PRESSO), we utilized the R package “MRPRESSO”, and for the robust adjustment contour score way (MR.RAPS), we used the R package “MR.raps”. We computed the statistical power of Mendelian randomization with the mRnd tool (accessible at https://cnsgenomics.shinyapps.io/mRnd/). Moreover, we used the PhenoScanner retrieval tool to estimate all the known phenotypes related with the genetic tools included in the exploration.

### Primary exploration

**Fig. 1:**
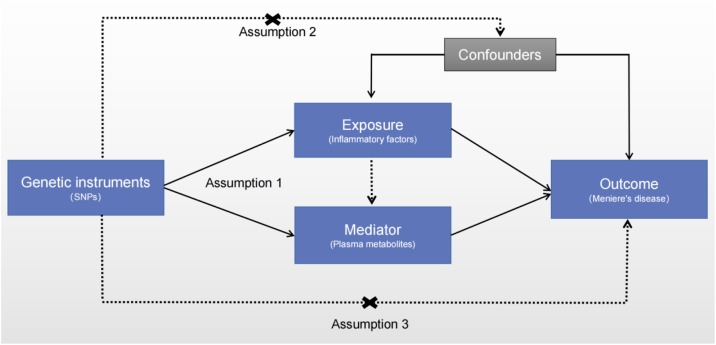
Three basic assumptions of genetic rate analysis and the schematic diagram of the design of the analysis method in this study. The basic assumptions of Mendelian randomization analysis include: Assumption 1 Correlation assumption, that is, the selected instrumental variable must have a significant correlation with the exposure factor; Assumption 2 Independence assumption, that is, the instrumental variable must have no significant correlation with potential confounding factors that may affect the exposure or outcome; Assumption 3 Exclusivity restriction, that is, the instrumental variable can only affect the outcome through the path of “instrumental variable → exposure factor → outcome”. SNP, single nucleotide polymorphism.

**Fig. 2:**
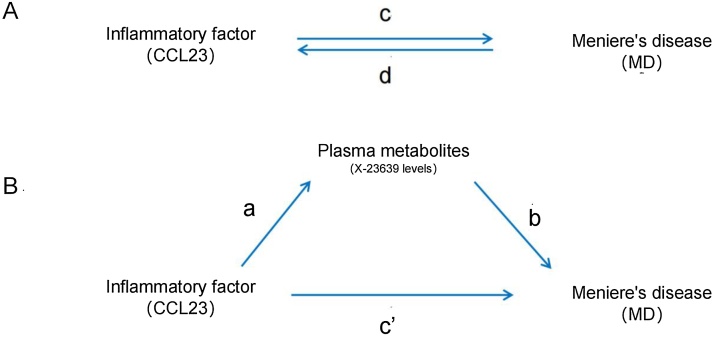
Diagrams illustrating the associations examined in this study. (A) Overall efficacy of Inflammatory Factors (IF) and Meniere's Disease (MD). c is the total effect of genetically predicted IF exposure and MD as a result. d is the total effect of using gene prediction of MD as exposure and IF as result. (B) Total effects are decomposed into: (i) Indirect effects, using a two-step method ([a] is the effect of IF on Plasma Metabolites [PM], [b] is the effect of PM on MD) and product method (a × b); (ii) Direct effect (c' = c-a × b). The mediated ratio is the indirect effect divided by the total effect.

Fig. 1 shows a diagram of the exploration process. Regarding the core terms of MR, we have provided detailed explanations in Supplementary Table S1. As shown in Fig. 2A, we used a two ‒ pattern biimmediateional MR way to study the two ‒ way causality between IF and MD. The overall impact was considered as the outcome. The IVW way uses meta ‒ exploration to combine the Wald ratios of the causal impact of each single SNP.^14^ Subsequently, the MR-Egger way and the weighted ‒ median way were utilized as supplementary ways to IVW.^15^^,^^16^ To acquire MR estimations, various ways tailored to different validity assumptions were put into use. The application of IVW hinges on the supposition that all SNPs serve as valid IVs. Thus, this way is capable of generating precise estimation outcomes. The MR-Egger way is utilized to evaluate the immediateional pleiotropy of IVs, with the intercept being interpretable as an approximation of the average pleiotropy of genetic variation. Unlike the MR-Egger exploration, the weighted ‒ median way is more precise, with a smaller standard deviation. In the case of horizontal pleiotropy, even if 50% of the genetic variants are invalid instrumental variables, the weighted ‒ median way can still offer consistent estimation consequences.^17^

### Mediation exploration

As shown in Fig. 2B, we carried out an extra mediating exploration using a two - step MR Design. Our aim was to find out if PM act as mediators in the causal path from IF to the MD outcome. The overall impact can be divided into a backhanded impact (achieved through intermediaries) and an immediate impact (happening without intermediaries).^18^ The overall influence of IF on MD can be broken down into two parts: (1) The immediate influence of IF on MD (marked as c' in Fig. 2B), and (2) The backhanded influence of IF through mediators (reappear as a × b in Fig. 2B). To figure out the proportion mediated by intermediate impact, we divide the backhanded impact by the total impact. At the same time, we computed the 95% Confidence Interval using the delta way.^19^

### Sensitivity exploration

We applied MR Steiger filtering to examine the causal direction of each selected SNP in relation to the exposure and the outcome.^20^ This method computes the variance that instrumental SNPs contribute to the exposure and the outcome. Subsequently, it determines if the variance in the outcome is lower than that in the exposure. If the MR Steiger analysis yields a “TRUE” result, it implies the causality is as anticipated. Conversely, a “FALSE” result indicates the causality is in the opposite direction. We excluded the SNPs with “FALSE” results since they suggest the SNP has a stronger effect on the outcome rather than the exposure.

We evaluated the heterogeneity among SNPs using Cochran’s *Q* statistic and funnel plots.^21^^,^^22^ We detected horizontal pleiotropy through the MR-Egger intercept method and the MRPRESSO method.^23^ When outliers were identified, we take away them and re-evaluated the MR causal appraise. If significant heterogeneity persisted after removal, we employed a random effects pattern to estimate the stability of the results. This pattern is less influenced by the weaker raletion between SNPs and the exposure. Finally, we conducted a leave-one-out analysis to determine the impact of each SNP on the overall causal appraise.

## Results

### Relation of IF with MD

After cyclic Mendelian randomization exploration of 91 IFs and MD, we screened IF (C-C motif chemokine 23 ranks, CCL23) with the lowest p-value and consistent beta immediateion as the best exposure factor. After eliminating the palindromic SNPs identified via MR Steiger filtering, ambiguous SNPs, SNPs lacking proxies, and SNPs with incorrect causal orientation, 32 SNPs in IF (CCL23) and 10 SNPs in MD were left as instrumental variables. As MD failed to achieve the genome ‒ wide marked rank of SNPs, SNPs without genome ‒ wide marked (p < 5 × 10^−6^) were utilized as IVs. Our research has a 100% capacity to detect a causal link between IF and MD risk.

**Fig. 3:**
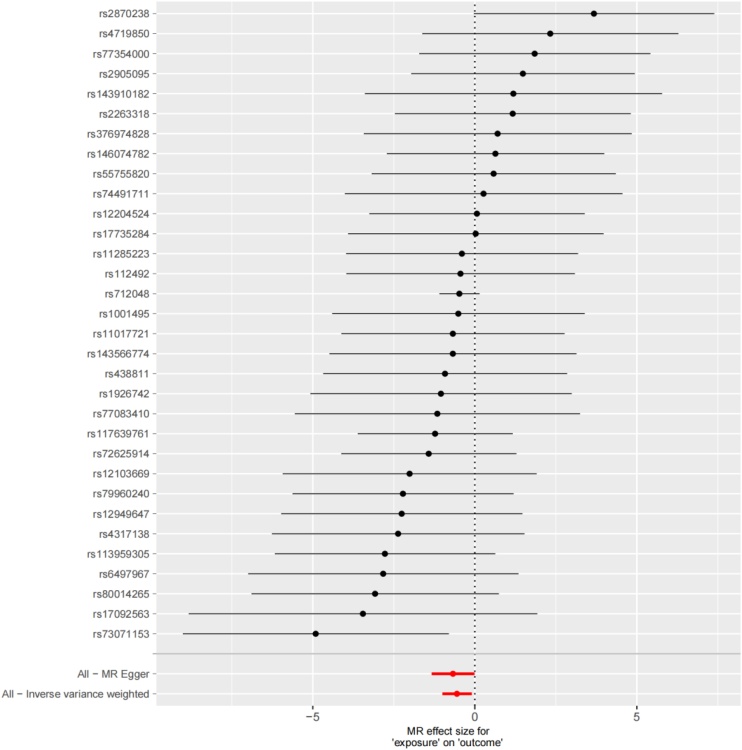
Forest plot to visualize causal effect of each single SNP on total MD risk.

**Fig. 4:**
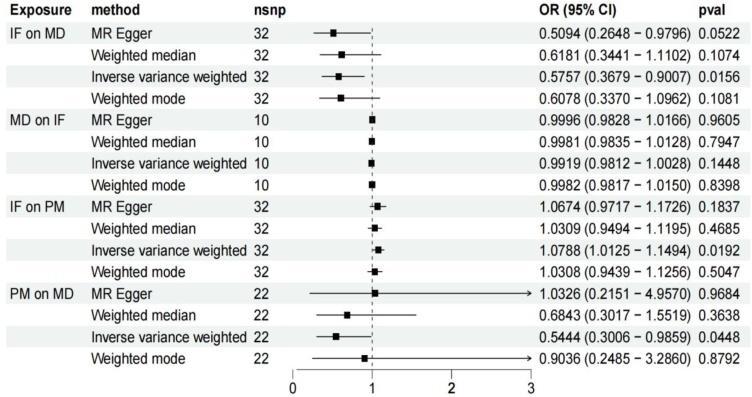
Forest plot to visualize the causal effects of PM with IF and MD.

The Inverse Variance Weighting (IVW), MR-Egger, and weighted median regression ways were employed to estimate the causality between genetically predicted IF and MD (Fig. 3, Fig. 4). Across all three MR ways, there was extensive and consistent evidence supporting a negative correlation between IF and MD (IVW Odds Ratio [OR] for per SD reduction in IF = 0.5757 [95% CI 0.3679‒0.9007], p = 0.0156; MR-Egger OR for per SD reduction in IF = 0.5094 [95% CI 0.2648‒0.9796], p = 0.0522; weighted median OR for per SD reduction in IF = 0.6181 [95% CI 0.3441–1.1102], p = 0.1074). Nevertheless, the consequences of our MR exploration indicated no causality from genetically predicted MD to IF. (That is, no causality from genetically predicted MD to IF.) The OR obtained by the IVW way was 0.9919 (95% CI 0.9812–1.0028; p = 0.1448). The consequence is appear in Fig. 4.

### Relation of IF with PM

We excluded palindromic and ambiguous SNPs, those lacking proxies, and SNPs with wrong causal immediateions determined by MR Steiger filtering. Then, 32 genome-wide marked SNPs were chosen as IVs. By using the IVW, MR-Egger, and weighted median ways, a positive correlation was found between genetically predicted IF and the risk of PM (X - 23639 rank). (The IVW way showed an OR of 1.0788; [95% CI 1.0125–1.1494], p = 0.0192. The MR-Egger way had an OR of 1.0674; [95% CI 0.9717–1.1726], p = 0.1837. The weighted median way had an OR of 1.0309; [95% CI 0.9494–1.1195], p = 0.4685.) The result is presented in Fig. 4.

### Relation of PM with MD

We display all the genetic tools related with PM at the genome ‒ wide marked threshold of p < 5 × 10^−8^. As shown in Fig. 4, there was a marked negative correlation between gene - predicted PM and MD. With the IVW way, the Odds Ratio (OR) was 0.5444, and the 95% Confidence Interval (95% CI) ranged from 0.3006 to 0.9859, with p = 0.0448. The IVW, weighted median, and weighted mode ways have the same estimation immediateions.

### Proportion of the relation between IF and MD mediated by PM

**Fig. 5:**
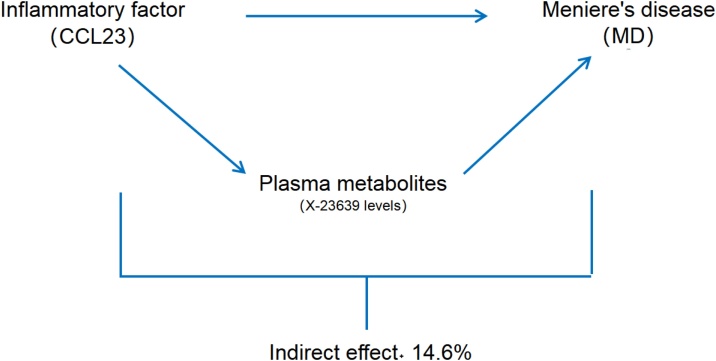
Schematic diagram of the PM mediation effect.

We examine PM as a mediator in the route from IF to MD. It was discovered that IF was linked to an increase in PM, and this, in turn, was related to a decreased risk of MD. As illustrated in Fig. 5, our research indicated that PM contributed to 14.6% of the reduction in MD risk related with IF (proportion mediated: 14.6%; 95%CI −0.76%‒1.05%).

### Sensitivity exploration

We carried out several sensitivity research to detect and correct potential pleiotropy in causal estimations. By using the Cochran's *Q* measurement and funnel plot, we found that there was no heterogeneity or asymmetry among these SNPs in terms of their causality (*Q* p-value > 0.05 and the funnel plot shows a normal distribution). In our research, the MR-Egger intercept didn't show any signs of pleiotropy at the immediateional rank of the IF instrument (p-value > 0.05). Moreover, the MR-PRESSO global measurement didn't detect potential horizontal pleiotropy (p-value = 0.736). We verified the impact of each SNP on the overall causal estimate through leave-one-out analysis. After removing each SNP, we conducted MR analysis on the remaining SNPs again. The results remained consistent, indicating that including all SNPs made the causality marked.

## Discussion

Our study employed a two-pattern, biimmediateional MR way to investigate the reciprocal causality between IFs and MD, with a particular focus on the function of Plasma Metabolites (PMs) as potential mediators. The discovery offers novel insights into the complex interplay between inflammation, metabolite ranks, and the pathogenesis of MD.

In our exploration, we identified C-C motif Chemokine 23 (CCL23) as the most marked IF related with MD risk. The MR exploration revealed a consistent negative correlation between genetically predicted CCL23 ranks and MD, indicating that higher ranks of CCL23 may be protective against MD. Conversely, we found no evidence of causality for genetically predicted MD influencing IF ranks, indicating a uniimmediateional relationship from IF to MD rather than vice versa.

MD, an inner ear disorder causing vertigo, tinnitus, hearing loss, and ear fullness, may link to CCL23 dysfunction despite lacking direct evidence.^24^ CCL23’s biological roles suggest mechanisms: 1) As a chemokine, excessive CCL23 could exacerbate inner ear inflammation by recruiting immune cells, worsening MD symptoms;^25^ conversely, reducing it might alleviate inflammation. 2) CCL23 supports cell proliferation and tissue repair^26^; its inhibition could impair inner ear regeneration, aggravating MD. 3) CCL23 mitigates endoplasmic reticulum stress and apoptosis; its suppression may increase cell death in MD.^27^ Additionally, elevated CCL23 might disrupt inner ear immune surveillance, akin to viral immune evasion. While no direct MD-CCL23 connection exists, its roles in inflammation, repair, and stress regulation imply potential involvement in MD pathogenesis.

Our mediation exploration further elucidated the causal pathway between IF and MD, revealing that PMs may mediate a portion of this relationship. Specifically, we found that genetically predicted IF ranks were related with increased PM ranks, which in turn were related with a reduced risk of MD. This indicates that PMs may act as intermediaries in the causal pathway from IF to MD, accounting for approximately 14.6% of the IF-related MD risk reduction. While the confidence interval for this proportion was wide (95% CI −0.76% to 1.05%), the immediateion of the impact was consistent across different MR ways, indicating a plausible mediating function for PMs.

In addition, according to the literature, betahistine and its metabolite 2-PAA have been found to act an vital function in the treatment of Meniere disease.^28^ Betahistine can improve the blood flow in the inner ear and adjust the histamine system, which can alleviate the symptoms of dizziness and help the vestibular function to recover slowly. The concentration of 2-PAA, the metabolite it produces in the body, in the blood can be used to judge whether the therapeutic impact of betahistine is good or not. Our study found that the plasma metabolite (the rank of X-23639) and the content of CCL-23 increased or decreased together, but it was inversely related to the severity of MD, that is, the more X-23639, the lighter MD might be. Therefore, the rank of X-23639 may also be used to judge MD. In order to ensure the reliability of the research consequences, we have done a lot of sensitivity exploration to see if there are some factors that will affect the consequences, such as pleiotropy and heterogeneity. With MR-Egger intercept measurement and MR-PRESSO global measurement, there is no immediateional pleiotropy or horizontal pleiotropy, which shows that the causality we have studied is unlikely to be inaccurate because of the impact of pleiotropy. In addition, through Cochran's *Q* measurement and funnel plot exploration, we found no heterogeneity or asymmetry among the SNPS used in the study, which further proves that the results of our study and exploration are reliable.

Our study has limitations despite robust findings. First, published GWAS data limit control over confounding factors and preclude individual-level analyses for deeper causal insights.^29^ Second, MR assumptions about SNPs as instrumental variables risk residual pleiotropy, necessitating alternative validation methods. Third, mediation analysis presumes PMs are true intermediaries between IFs and MD; sensitivity analyses support this, but experimental confirmation is needed. Finally, the genome-wide association study data based on the European population has limitations and cannot be universally applicable. In the future, more samples need to be included to conduct diverse population studies to verify the consistency across different races.

## Conclusion

Our study reveals novel bidirectional causality between IFs, PMs, and MD, with elevated CCL23 levels potentially protective against MD through partial PM mediation. These findings emphasize the inflammation-metabolism interplay in MD pathogenesis and highlight therapeutic targets. Future research should validate results across diverse populations and explore underlying biological mechanisms to advance targeted treatments.

## ORCID ID

Jian-Dao Hu: 0009-0009-5992-3268

Jing Qian: 0009-0007-2364-0544

## Funding

The Zhejiang Province Traditional Chinese Medicine Science and Technology Plan Project (2025ZX165); Yinzhou District Health Science and Technology Program (2024Y01).

## Declaration of competing interest

The authors declare that the research was conducted in the absence of any commercial or financial relationships that could be construed as a potential conflict of interest.
