# Supplementary material for: Genetically predicted plasma metabolites mediate the relation between inflammatory factors and Meniere's disease
Source: Braz J Otorhinolaryngol. 2026 Feb 5;92(2):101772. doi: 10.1016/j.bjorl.2026.101772 (PMC12906162; doi:10.1016/j.bjorl.2026.101772)
Supplement: Supplementary file 1 [file mmc1.docx]

**BJORL-D-25-00111_ Supplementary Material**

**Supplementary Table 1** Mendelian Randomization is a method that uses genetic variations to study the causal relationship between exposure factors and health outcomes. Its core terms include.

| **Instrumental Variable** | Instrumental Variable refers to genetic variations (usually single nucleotide polymorphisms, SNP) that are associated with specific exposure factors (such as lifestyle or biomarkers). These instrumental variables are used to assess the causal relationship between exposure factors and health outcomes (such as disease occurrence or mortality). In general, in Mendelian randomization, genetic variations are used as instrumental variables. |
| --- | --- |
| **Exposure Factors** | In clinical medicine, Exposure Factors refer to any factors that may affect health, the occurrence of diseases, or pathological conditions. These factors can be biological, chemical, physical, socio-economic, or lifestyle related. Exposure factors can be associated with an individual's health status and influence the risk and development of diseases. |
| **Confounding factors** | In medical research, Confounding Factors refer to those variables that may affect the research results. These variables are related to both the main exposure factor and the outcome variable, and may lead to misunderstandings or biases regarding the causal relationship. The presence of confounding factors may prevent researchers from accurately determining the true relationship between the exposure (such as a certain treatment, lifestyle or environmental factor) and the outcome (such as disease occurrence, mortality rate). |
| **Outcome Variables** | In Mendelian Randomization studies, the “Outcome” typically refers to the health outcomes or disease states that are of interest in the research. The outcome can be various different indicators and is usually evaluated in terms of its causal relationship with the exposure factors (such as lifestyle, environmental factors, or biomarkers). |
| **Causal relationship** | Altering the exposure level will change the outcome (or the risk of the outcome). “Association” does not necessarily imply a causal relationship; it merely indicates that there is a correlation between the exposure level and the outcome. |
| **Confusion** | Confusion refers to the distortion that occurs in the estimation of the relationship between a risk factor and the outcome when the risk factor is associated with another factor that also influences the outcome. For example, the association between alcohol consumption and the risk of coronary heart disease may be confused by the fact that people who drink alcohol are also more likely to smoke, which has a causal impact on the disease risk. |
| **Genome-Wide Association Study (GWAS)** | An hypothesis-free research design that tests the association between thousands or millions of genetic variations and phenotypes. The main purpose of GWAS is to identify the variations related to the phenotype, which can be used to identify the genes related to the cause of the phenotype or to develop predictive polygenic scores for the phenotype. |
| **Linkage Disequilibrium (LD)** | LD refers to the non-independent segregation of genetic variations. Genetic variations located close to each other on the same chromosome can be inherited together. If the allele frequencies are similar, this may lead to a correlation between them. |
| **Multifactoriality** | Multifactoriality refers to the association of a genetic variation with multiple phenotypes. Horizontal multifactoriality refers to a genetic variation being associated with more than one phenotype through distinct biological pathways. This type of multifactoriality is concerning because it violates the exclusion restriction assumption and may distort the results. Vertical multifactoriality refers to the association of a genetic variation with more than one phenotype within the same biological pathway, and this does not render the research results invalid. |
| **Reverse causality** | A situation where a result (disease) affects the exposure level (risk factor) rather than the other way around, as expected. This bias is minimized in MR studies because genetic variations are immutable and not influenced by the disease state. |
| **Single nucleotide polymorphism** | A common genetic variation, in which a base in the DNA has undergone a change (for example, a C instead of a T at a specific position in the gene sequence). |
| **Inverse variance weighting method** | The most effective (with the greatest statistical power), and is usually the main analytical method in MR studies that involves multiple genetic variations. It requires that all genetic variations be valid instrumental variables. |
| **Weighted median** | A common complementary method in MR studies, it is operated by taking the median of the variant specificity estimates. It is robust to outliers, but sensitive to the addition or removal of genetic variations. |
| **Multivariate MR** | A statistical method that enables the incorporation of genetic variations associated with multiple risk factors into the analysis. This method can be used to adjust for known confounding factors or to explore the mediating role of factors in the causal pathway from the risk factors of interest to the outcome. |
| **MR-egger** | MR is a common supplementary method in the MR study. It can test and adjust multi-effectivity, but it is sensitive to outliers and has lower efficiency compared to the inverse variance weighting method. |
| **MR-PRESSO** | It can identify and remove outliers, but it has a high false positive rate and several effective instrumental variables. |
